# Automatic quality measurement of aortic contrast-enhanced CT angiographies for patient-specific dose optimization

**DOI:** 10.1007/s11548-020-02238-4

**Published:** 2020-07-31

**Authors:** René Pallenberg, Marja Fleitmann, Kira Soika, Andreas Martin Stroth, Jan Gerlach, Alexander Fürschke, Jörg Barkhausen, Arpad Bischof, Heinz Handels

**Affiliations:** 1 Institute of Medical Informatics, University of Lübeck, Ratzeburger Allee 160, 23562 Lübeck, Germany; 2 Department of Radiology and Nuclear Medicine, UKSH Lübeck, Ratzeburger Allee 160, 23538 Lübeck, Germany; 3 IMAGE Information Systems Europe, Lange Str. 16, 18055 Rostock, Germany

**Keywords:** CT angiography, Rule-based classification, Template matching, Personalized healthcare, Automatic ROI detection

## Abstract

**Purpose:**

Iodine-containing contrast agent (CA) used in contrast-enhanced CT angiography (CTA) can pose a health risk for patients. A system that adjusts the frequently used standard CA dose for individual patients based on their clinical parameters can be useful. As basis the quality of the image contrast in CTA volumes has to be determined, especially to recognize excessive contrast induced by CA overdosing. However, a manual assessment with a ROI-based image contrast classification is a time-consuming step in everyday clinical practice.

**Methods:**

We propose a method to automate the contrast measurement of aortic CTA volumes. The proposed algorithm is based on the mean HU values in selected ROIs that were automatically positioned in the CTA volume. First, an automatic localization algorithm determines the CTA image slices for certain ROIs followed by the localization of these ROIs. A rule-based classification using the mean HU values in the ROIs categorizes images with insufficient, optimal and excessive contrast.

**Results:**

In 95.89% (70 out of 73 CTAs obtained with the ulrich medical CT motion contrast media injector) the algorithm chose the same image contrast class as the radiological expert. The critical case of missing an overdose did not occur with a positive predicative value of 100%.

**Conclusion:**

The resulting system works well within our range of considered scan protocols detecting enhanced areas in CTA volumes. Our work automized an assessment for classifying CA-induced image contrast which reduces the time needed for medical practitioners to perform such an assessment manually.

## Introduction

Visualizing blood vessels for image diagnostics and interventional therapy in radiological examinations is a non-negligible task carried out by performing a CT angiography (CTA). The CTA uses iodine-containing contrast agent (CA) to highlight target areas for the detection of pathological findings like aneurysms and aortic dissections [8]. However, the iodine contained in the CA can cause delayed allergic reactions, cutaneous reactions and contrast-induced nephropathy [1]. To reduce those risks patient-individual CA dose adjustments would be preferable over using a standard dose as in current clinical routine examinations. The adjustments could minimize overdosing the CA or generating images with insufficient contrast caused by a too small CA dose. To determine the dependencies between patient-individual vital parameters and the CA dose the CTA image contrast induced by the CA has to be assessed. A manual assessment of the image contrast conducted by experts that includes the selection and determination of regions of interest (ROI) and a classification of the mean Hounsfield units (HU) would be not-workable in clinical routine.

**Fig. 1:**
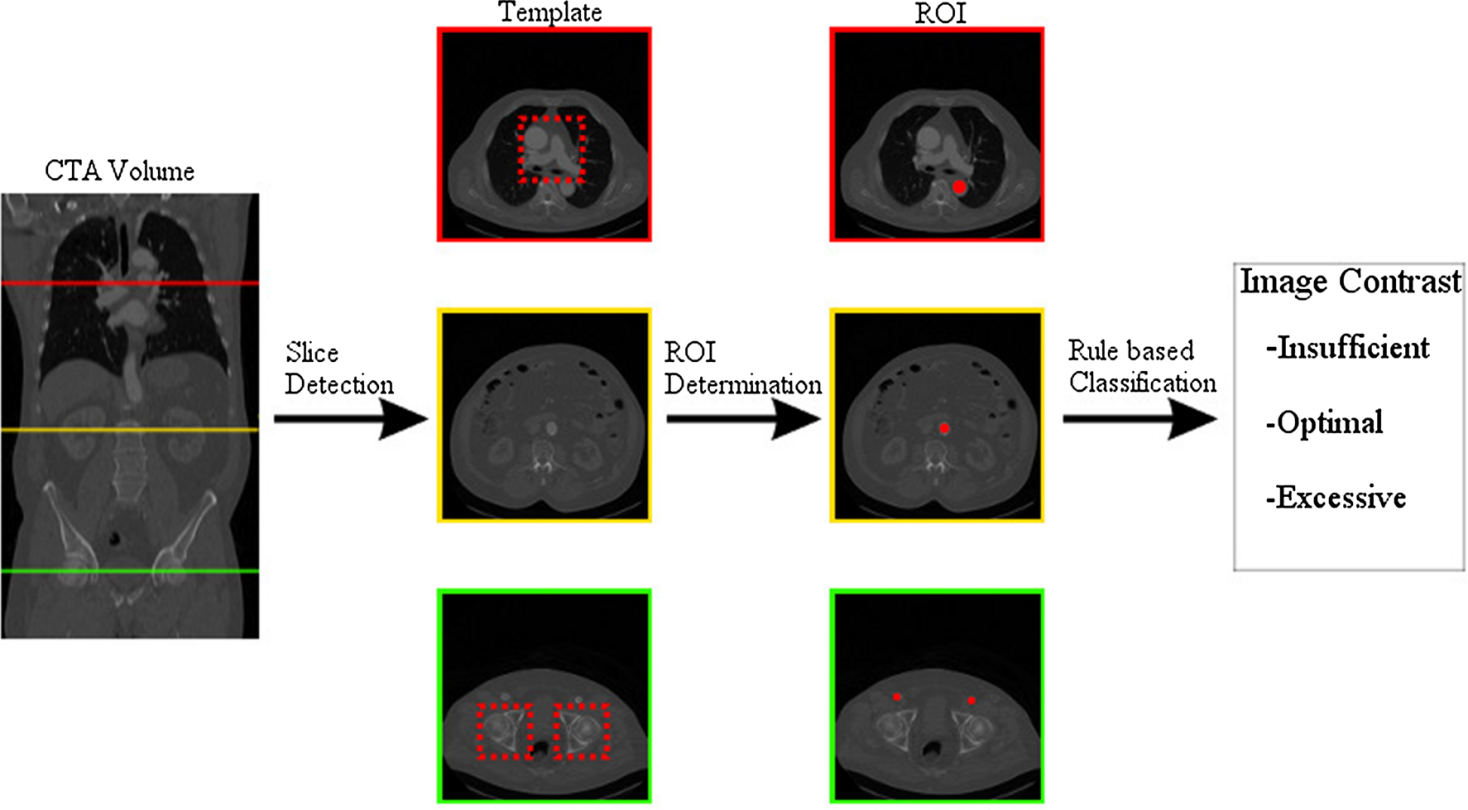
This graphic gives an overview of the three major components of the automatic image contrast measurement process

**Fig. 2:**
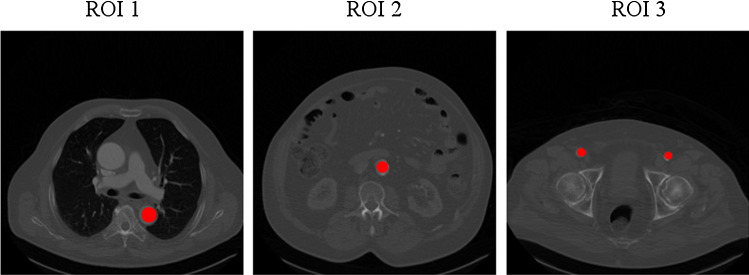
Example of positioning ROI 1, 2 and 3 in the aorta and the arteria femoralis communis, respectively

To circumvent the time-consuming task of assessing the image contrast by experts we propose a method for an automatic image quality measurement of aortic CTA volumes. The assessment is implemented as a rule-based classification based directly on the HU values of 2D ROIs distributed over the CTA volume. Our approach consists of three consecutive components as shown in Fig. 1. The first step is to detect the slices suitable for the ROIs. We applied a template matching as this method is a staple in medical image processing and showed success in various use cases [5, 7]. Following this is the automatic ROI determination. The ROIs are positioned in the aorta and the arteria femoralis communis, respectively, and are determined by combining the circle Hough transform [3] and the vessel filter [6] for smaller structures. Taking the mean HU of each ROI a rule-based approach was applied. The rules were defined by radiological experts to assign the data set to one of the following three classes: insufficient image contrast, optimal image contrast or excessive image contrast.

## Methods

In the standard procedure for contrast measurement for aortic CTA volumes three ROIs were manually positioned in axial CTA slices by radiologic experts (Fig. 2):

- *ROI 1* Aorta, level: pulmonary artery bifurcation level.
- *ROI 2* Aorta, level: kidneys.
- *ROI 3* Arteria femoralis communis.

The ROIs were defined by the radiological experts of our team who were blinded to the to be labeled patients. The ROIs lie in axial slices which contain concise anatomical landmarks to provide good starting conditions for the proposed automatization. The rule-based classification assigns one of three classes, insufficient, optimal or excessive contrast to a patients CTA volume. The mean HU values of ROI 1, 2 and 3 were taken and each was assigned into one of the categories as presented in Table 1.

**Table 1:** Expert contrast quality categorization based on the HU values of the ROIs

Category	Range [HU]	Definition
A	$\le 180$	HU value too low
B	181–240	Lower tolerance area
C	241–300	Target area
D	301–360	Upper tolerance area
E	$>360$	Excessive HU value

The thresholds of the categories were chosen according to radiological experts and their usage in literature (e.g., [2]). After the assignment of categories, rules were formulated by the experts to classify each combination of categories. As presented in Table 2 one of three classes could be assigned. With class 3 representing excessive image contrast which can be associated with an CA overdosing. CTA volumes of class 1 show insufficient image contrast which in turn can be a result of, e.g., a too small CA dose and timing problems of the CT scan. If only two ROIs are available the rules are adapted accordingly.

**Table 2:** This table includes the class division based on the expert categorization in Table 1

Class	Image contrast	Condition
1	Insufficient	At least one ROI is inA
2	Optimal	All ROIs in category B, C and D
3	Excessive	At least one ROI in E, remaining in D

In the following chapters, a method for the automatic determination of ROI 1, 2 and 3 is presented for the image contrast measurement.

### Automatic slice detection

**Fig. 3:**
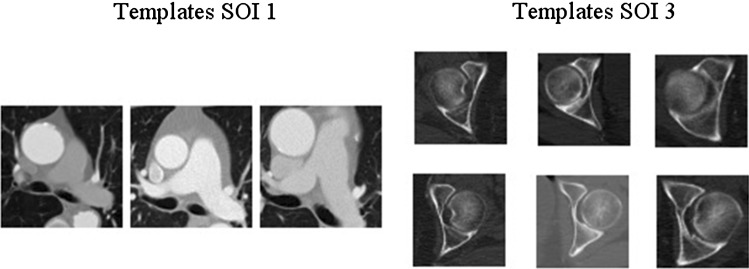
Example templates for the detection of SOI 1 and 3

The first step of the automatic assessment is the detection of a suitable axial slice of interest (SOI) for each ROI. The aorta and the artery do not have a generalized shape for patients undergoing a CTA examination. Therefore, the algorithm is searching for the following significant anatomical structures (Fig. 3) which are located on the corresponding slices of the ROIs:

- *SOI 1* Pulmonary artery bifurcation for ROI 1.
- *SOI 2* Mean of SOI 1 and 3 for ROI 2.
- *SOI 3* Caput ossis femoris for ROI 3.

For detecting the aforementioned structures in ROI 1 and 3 we applied a template matching approach [5]. The templates were extracted as 2D image patches from the CTA volumes. Before matching the spacing of the templates was adjusted to the CTA data of the assessed patient. The search space was chosen as a subset of all slices in the considered CTA data to optimize the computational demand. To determine SOI 1 the search space was limited to the upper two thirds of axial slices. For SOI 3 the lower two thirds were chosen and at least two templates were used containing the left and right femoral head as a set extracted from the same reference data. The template matching algorithm was applied to each slice of the search space and the value of the similarity function was recorded.

**Fig. 4:**
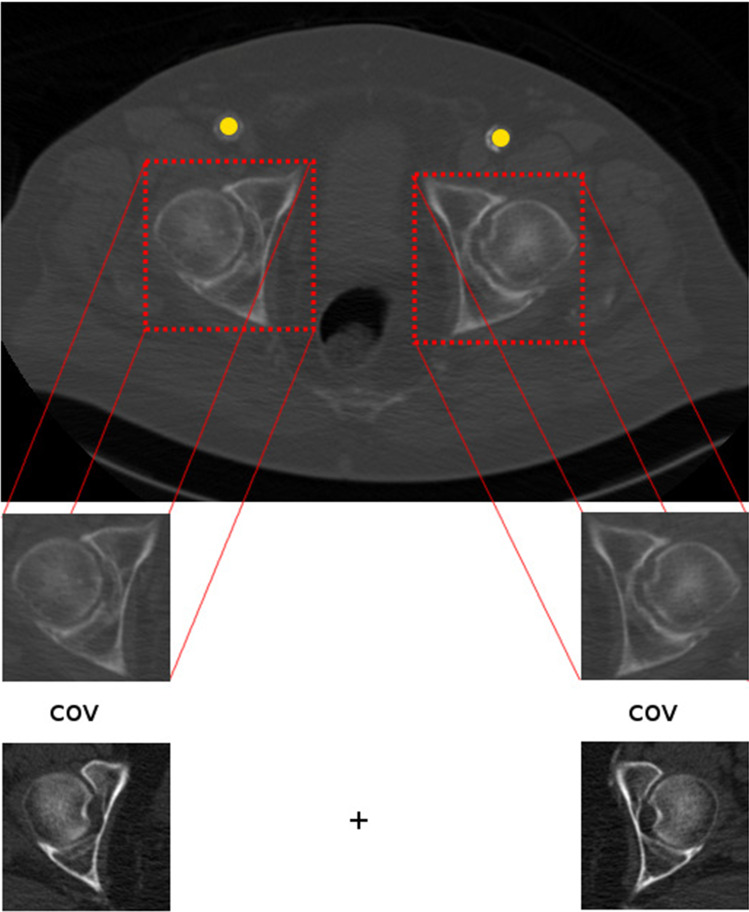
This figure visualizes how the template matching detects the caput femoris in the CT slice

As the HU values in CTA volumes can show a high degree of variability in the artery a normalized cross-correlation measure was implemented as similarity function between the template and the image patches of the considered axial slice extracted in a sliding-window manner. The slice that resulted in the highest value of the similarity function was determined as the SOI regardless of the number of used templates or in case of SOI 3 the number of sets of templates. To measure the similarity for the determination of SOI 3 a set of templates was defined and their mean of their individually obtained cross-correlation values was computed (Fig. 4).

The slice determination of SOI 2 forms an exception. Because of the high variability of the anatomical structures in the abdomen, the appropriate slice for SOI 2 in the standard case is chosen as the mean of the determined slices of SOI 1 and 3. In a few exceptional cases of scans, the CTA volumes do not contain the axial slices suited for SOI 1 or 3. This causes a problem for the aforementioned determination of SOI 2. It must therefore first be established whether the required slices for SOI 1 and 3 exist. Based on the similarity value of the determined slice taken from the template matching a threshold was empirically defined. If the similarity value is below 0.6 the algorithm declares a SOI as non-existing. The algorithm then uses an offset applied to the existing SOI (SOI 1 or SOI 3) to determine SOI 2. The offset was chosen as the halved average distance between ROI 1 and 3 of the expert-annotated axial slices. For our data the offset was therefore 46 slices or 23 cm based on the constant z-spacing of 5 mm.

### Automatic ROI determination

After all available SOIs are determined the algorithm proceeded with the determination of ROI 1, 2 and 3. Because of the generally round shape of the aorta in the axial plane the circle Hough transform was applied to determine ROI 1 and 2. The smaller size of the arteria femoralis communis leads to the decision to use the vesselness filter [6] for the determination of ROI 3.

By applying the circle Hough transform the CTA image slice is transformed into a Hough space represented as an accumulator matrix parameterized by the radius and the coordinates of the circle center. As the aorta is not the only circular structure (e.g., the vertebral body) a dynamic programming approach [4] computing a cost function for the selection of the most probable aorta was implemented. The six highest accumulations in the Hough space are taken as candidates for the aorta and were used as starting point for the calculation of the cost function. The costs for final circle candidate $x_k$ with $k = 1,\ldots , 6$ is $C(x_k ) = \sum _{i}^{i+4} C(x_{ki})$ , (*i* being the according SOI) cumulates the costs of possible circles over adjacent axial slices. The individual cost for each considered circle is defined as followed:

1 $$\begin{aligned} C(x_{ki})=C_\mathrm{v}(x_{k,i})+C_\mathrm{r}(x_{k,i-1},x_{ki})+C_\mathrm{d}(x_{k,i-1},x_{ki}) \end{aligned}$$

where $C_\mathrm{v}(x_{ki})$ describes the variance within the circle $x_{ki}$. $C_\mathrm{r}(x_{k,i-1},x_{ki})$ describes the radius difference of two circles between slice $i - 1$ and *i* and $C_\mathrm{d}(x_{k,i-1}, x_{ki} )$ describes the distance of two circles centers of two adjacent slices. The starting circle of the combination of circles is selected that appears the most constant over the five considered slices resulting in the lowest overall cost. To avoid calcification on the vascular walls the circle radius is reduced by 4 pixels. If no circle achieves a score under 200 no circle will be selected which implies that the aorta will not be detected and has to be determined by the medical practitioner.

Because of the lower diameter of the arteria femoralis communis after applying the vesselness filter to the SOI 3 a thresholding is applied to exclude bone material and calcification. The algorithm returns ROI 1, 2 and 3 as input for the concluding assessment.

## Evaluation and results

### Data

Our data consist of 73 contrast-enhanced CTA volumes that can contain pathological findings. These images were generated at the Department of Radiology and Nuclear Medicine located at the UKSH Lübeck.The Siemens SOMATOM Definition AS+ and the ulrich medical CT motion contrast media injector with RIS/PACS interface were used. The administered iodine containing CA dose was 100 ml. As CA Imeron was used at a concentration of 300 mg/ml, it was administered with an injection rate of 5 ml/s.

The volumes have been generated using an aorta scan protocol. The z-spacing is 5 mm for all volumes with varying spacing for the remaining dimensions. All axial slices have a resolution of $512\times 512$ voxel. The image data and the corresponding metadata were stored in the DICOM format. For each CTA volumes radiological experts annotated the SOIs, the ROIs and the image contrast quality class (Table 2). These annotations provide the reference standard for the evaluation of our provided automatic image contrast measurement.

### Automatic slice detection

To increase the generalization of the template matching we created a pool of possible templates taken from our CTA volumes. We tested each combination of templates for the number of templates $t = 1, \ldots ,5$. As a mean to evaluate the templates and the slice detection we computed the absolute difference in slices between the predicted SOI and the slice chosen by the radiological expert. Our best results shown in Table 3 were achieved by a combination of $t = 3$ templates for each SOI.

**Table 3:** Results of the slice detection for the three ROIs

Slice difference	0–2	3–5	5–10	> 10
SOI 1	56	7	3	2
SOI 2	27	28	9	2
SOI 3	48	9	1	0

The table describes the number of occurrences of a particular bin of slice difference. The absolute difference in slices is calculated between the predicted slice and the expert selection. Note that not every SOI exits in every CTA volume hence the varying total number of occurrences

**Fig. 5:**
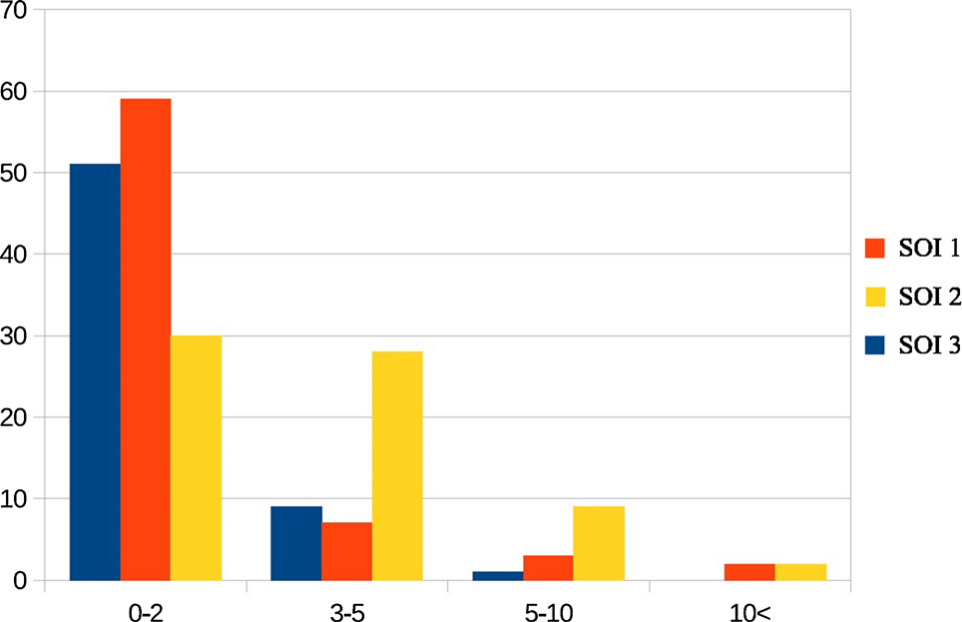
Histogram of the difference between predicted slices and expert annotations for the three ROIs

For the validation the CTA volumes from which the templates were taken, were omitted for calculating the results. Figure 5 visualizes the corresponding results. The algorithm detected 56 out of 68 slices for SOI 1 with a only an detection error of max. 2 slices implying that 82.35% were detected within max. 10 mm deviation of the expert’s chosen slice. For SOI 3 the corresponding rate was 82.76%. For SOI 2 40.90% of the detected slices were deviating up to two slices, but regarding a slightly higher max. deviation up to 5 slices or 25 mm still 83.33% fell into this category.

In addition the algorithms ability to confirm or deny the existence of a slice that would be considered as the SOI in the current CTA volume is evaluated. Table 4 shows the number of missing and available slices of SOI 1 and 3 in comparison with the experts choice. The algorithm achieved a sensitivity and specificity of 100% for SOI 1 and a sensitivity of 100% and a specificity of 83.33% for SOI 3. This shows that the algorithm is capable to a rather high degree.

**Table 4:** The two fourfold tables show the results of the algorithms existence check for SOI 1 and 3 in comparison to the experts opinion

		Expert slice	
		Available	Missing
SOI 1	Available	71	0
	Missing	0	2
SOI 3	Available	61	2
	Missing	0	10

### Automatic ROI determination

For the evaluation of the ROI determination three possible outcomes of the segmentation were considered. “Correct” was recorded when the ROI was placed within the defined vessel. “Not found” corresponds to the circle conditions (“Automatic ROI determination” section) not being met leading to no set ROI. If a wrong circular anatomical structure (e.g., the left ventricle) was determined as the ROI we assigned the label “incorrect.” For ROI 1 our algorithm reached 78.87% accuracy locating the aorta. An accuracy of 60.67% was achieved for ROI 2 reflecting the higher occurrence of pathological findings like aneurysms and dissections in the abdomen area deforming the overall shape of the aorta. The low accuracy 4.9% for ROI 3 shows that the vesselness filter was the wrong choice and will be replaced in further research. The detailed results can be found in Table 5.

**Table 5:** Resulting distribution of the automatic determination for each ROI

	Correct	Not found	Incorrect
ROI 1	56	10	5
ROI 2	46	18	5
ROI 3	3	15	43

Note that not every CTA volume contains all three SOIs which leads to some scans only containing two ROIs hence the varying total number for each row

### Rule-based classification

At last, the extent to which the classification of the image contrast results corresponds with the classes the experts chose for our aortic CTA volumes has to be evaluated. In Table 6 are the results of the algorithms results presented in comparison to the experts ones. In 95.89% of the cases our quality measurement chose the same image contrast class. This corresponds to 70 out of 73 CTA volumes. An additional positive note is that if a CTA volume is misclassified the deviation to the correct class is only 1 class.

Considering our focus on potentially giving recommendations for a CA dose reduction the system should be particularly skilled at identifying class 3 cases. We analyzed this scenario by merging class 1 and 2 together to calculate the positive predictive value of class 3 on the resulting two-class problem. This resulted in a positive predictive value of 100% meaning that all cases classified as class 3 by the system actually belong to class 3.

**Table 6:** This table shows the compression between the reference assessment and the assessment using predicted slices

		Expert			
		1	2	3	
	1	3	0	0	3
Predict	2	1	15	2	18
	3	0	0	52	52

## Discussion and conclusion

In an effort to form the basis for a patient-individual CA dose adjustment we proposed a method to automize an image contrast measure of aortic CTA volumes which otherwise was conducted manually by radiological experts. Our algorithm assesses the volumes and classifies the image contrast based directly on mean HU values of predefined ROIs. The slice detection implemented with a template matching approach as a first step worked rather well and in further experiments we will include a test set of new CTA volumes to improve the generalizability and its verification. The segmentation of the ROIs achieved varying results with the accuracy of ROI 3 clearly indicating the use of another segmentation method in future research. In contrast to this the results for ROI 1 and 2 showed good generalization in some cases even with major appearance of deforming pathologies. The concluding evaluation of the rule-based classification showed that our algorithm is capable of performing the image contrast measurement automatically. The time measurement has only taken place qualitatively resulting in an approximate reduction in the time needed to assess on CTA volume by one third when a rectifying action is required. In the percentage of cases where the algorithm is already working completely automatically it is faster by approximately 80%.

With our focus on reducing a CA dose we are aware of the limitations regarding the insufficient image contrast class. Statements as to whether the dose was too low are not unambiguously applicable, since there are variety of reasons such as timing problems during the CT scan or a low cardiac output. For our ongoing research we will lay the focus on expanding the assessment to other scan protocols like pulmonal scans to easily extend the amount of data that can be used for further purposes.
